# 睾丸弥漫大B细胞淋巴瘤的临床病理特征和预后分析

**DOI:** 10.3760/cma.j.issn.0253-2727.2023.04.010

**Published:** 2023-04

**Authors:** 玥 王, 子旸 石, 晴 施, 硕 王, 慕晨 张, 容 沈, 洋 赫, 惠玲 裘, 红梅 易, 磊 董, 黎 王, 澍 程, 彭鹏 许, 维莅 赵

**Affiliations:** 上海交通大学医学院附属瑞金医院血液科，医学基因组学国家重点实验室，上海血液学研究所，上海 200025 State Key Laboratory of Medical Genomics, Shanghai Institute of Hematology, Shanghai Rui Jin Hospital, Shanghai Jiao Tong University School of Medicine, Shanghai 200025, China

**Keywords:** 睾丸, 淋巴瘤，大B细胞，弥漫性, 病理学，临床, 基因表达谱, 预后, Testis, Lymphoma, large B-cell, diffuse, Pathology, clinical, Gene expression profiling, Prognosis

## Abstract

**目的:**

探讨睾丸弥漫大B细胞淋巴瘤（DLBCL）的临床病理特征及预后。

**方法:**

回顾性分析2001年10月至2020年4月上海交通大学医学院附属瑞金医院收治的68例睾丸DLBCL患者的临床病理资料，采用靶向测序（55个淋巴瘤相关基因）评估患者的基因突变情况，同时进行生存和预后因素分析。

**结果:**

68例睾丸DLBCL中，原发睾丸DLBCL患者45例（66.2％），继发睾丸DLBCL患者23例（33.8％）。继发睾丸DLBCL患者Ann Arbor分期Ⅲ～Ⅳ期（*P*<0.001）、LDH升高（*P*<0.001）、ECOG评分≥2分（*P*＝0.005）、IPI评分3～5分（*P*<0.001）的比例高于原发睾丸DLBCL患者。62例（91％）患者接受以R-CHOP（利妥昔单抗+环磷酰胺+阿霉素+长春新碱+泼尼松）方案为基础的治疗，54例（79％）患者在化疗前接受睾丸切除术。继发睾丸DLBCL患者的预期5年无进展生存（PFS）率（16.5％对68.1％，*P*<0.001）及预期5年总生存（OS）率（63.4％对74.9％，*P*＝0.008）低于原发睾丸DLBCL患者，继发睾丸DLBCL患者的完全缓解率（57％对91％，*P*＝0.003）也低于原发患者。ECOG评分≥2分（PFS：*P*＝0.018；OS：*P*<0.001）、Ann Arbor分期Ⅲ～Ⅳ期（PFS：*P*<0.001；OS：*P*＝0.018）、LDH升高（PFS：*P*＝0.015；OS：*P*＝0.006）、多结外受累（PFS：*P*<0.001；OS：*P*＝0.013）是睾丸DLBCL患者的不良预后因素。20例睾丸DLBCL患者的靶向测序结果显示，突变频率≥20％的突变基因为PIM1（12例，60％）、MYD88（11例，55％）、CD79B（9例，45％）、CREBBP（5例，25％）、KMT2D（5例，25％）、ATM（4例，20％）、BTG2（4例，20％），继发睾丸DLBCL患者KMT2D突变发生率高于原发睾丸DLBCL患者（66.7％对7.1％，*P*＝0.014），且与睾丸DLBCL患者较低的5年PFS率相关（*P*＝0.019）。

**结论:**

继发睾丸DLBCL患者的PFS和OS较原发睾丸DLBCL患者更差。ECOG评分≥2分、Ann Arbor分期Ⅲ～Ⅳ期、LDH升高和多结外受累为睾丸DLBCL的不良预后因素。PIM1、MYD88、CD79B、CREBBP、KMT2D、ATM、BTG2是睾丸DLBCL中常见的突变，KMT2D突变患者预后不佳。

## Abstract (en)

**Objective:**

To analyze the clinicopathologic characteristics and prognosis of testicular diffuse large B-cell lymphoma（DLBCL）.

**Methods:**

A retrospective analysis was performed on 68 patients with testicular DLBCL admitted to Ruijin Hospital affiliated to Shanghai Jiao Tong University School of Medicine from October 2001 to April 2020. The gene mutation profile was evaluated by targeted sequencing（55 lymphoma-related genes）, and prognostic factors were analyzed.

**Results:**

A total of 68 patients were included, of whom 45（66.2％）had primary testicular DLBCL and 23（33.8％）had secondary testicular DLBCL. The proportion of secondary testicular DLBCL patients with Ann Arbor stage Ⅲ–Ⅳ（*P*<0.001）, elevated LDH（*P*<0.001）, ECOG score ≥ 2 points（*P*＝0.005）, and IPI score 3–5 points（*P*<0.001）is higher than that of primary testicular DLBCL patients. Sixty-two（91％）patients received rituximab in combination with cyclophosphamide, adriamycin, vincristine, and prednisone（R-CHOP）-based first-line regimen, whereas 54 cases（79％）underwent orchiectomy prior to chemotherapy. Patients with secondary testicular DLBCL had a lower estimated 5-year progression-free survival（PFS）rate（16.5％ *vs* 68.1％, *P*<0.001）and 5-year overall survival（OS）rate（63.4％ *vs* 74.9％, *P*＝0.008）than those with primary testicular DLBCL, and their complete remission rate（57％ *vs* 91％, *P*＝0.003）was also lower than that of primary testicular DLBCL. The ECOG scores of ≥2（PFS: *P*＝0.018; OS: *P*<0.001）, Ann Arbor stages Ⅲ–Ⅳ（PFS: *P*<0.001; OS: *P*＝0.018）, increased LDH levels（PFS: *P*＝0.015; OS: *P*＝0.006）, and multiple extra-nodal involvements（PFS: *P*<0.001; OS: *P*＝0.013）were poor prognostic factors in testicular DLBCL. Targeted sequencing data in 20 patients with testicular DLBCL showed that the mutation frequencies of ≥20％ were PIM1（12 cases, 60％）, MYD88（11 cases, 55％）, CD79B（9 cases, 45％）, CREBBP（5 cases, 25％）, KMT2D（5 cases, 25％）, ATM（4 cases, 20％）, and BTG2（4 cases, 20％）. The frequency of mutations in KMT2D in patients with secondary testicular DLBCL was higher than that in patients with primary testicular DLBCL（66.7％ *vs* 7.1％, *P*＝0.014）and was associated with a lower 5-year PFS rate in patients with testicular DLBCL（*P*＝0.019）.

**Conclusion:**

Patients with secondary testicular DLBCL had worse PFS and OS than those with primary testicular DLBCL. The ECOG scores of ≥2, Ann Arbor stages Ⅲ–Ⅳ, increased LDH levels, and multiple extra-nodal involvements were poor prognostic factors in testicular DLBCL. PIM1, MYD88, CD79B, CREBBP, KMT2D, ATM, and BTG2 were commonly mutated genes in testicular DLBCL, and the prognosis of patients with KMT2D mutations was poor.

非霍奇金淋巴瘤（NHL）是一种淋巴系统的异质性恶性肿瘤[1]。睾丸弥漫大B细胞淋巴瘤（DLBCL）是一种罕见的侵袭性结外NHL[2]，占NHL病例的1％～2％，可分为原发和继发两类[3]–[4]。以睾丸肿块为原发症状或主要症状，无明显其他结外器官受累，经病理确诊为DLBCL的患者为原发睾丸DLBCL[3],[5]。原发于其他部位，系统性累及睾丸的DLBCL为继发睾丸DLBCL[3],[5]。目前，睾丸DLBCL的文献报道较少，发病机制未明，综合治疗（手术联合免疫化疗、放疗、鞘内注射）后仍存在较高的结外复发风险[6]–[7]。本研究回顾性分析了上海交通大学医学院附属瑞金医院收治的68例睾丸DLBCL患者，旨在对睾丸DLBCL的临床及病理特征、基因突变谱及相关预后因素进行探究，为临床实践提供参考。

## 病例与方法

1. 病例：将2001年10月至2020年4月上海交通大学医学院附属瑞金医院收治的68例初治睾丸DLBCL患者纳入本研究（其中45例为原发睾丸DLBCL，23例为继发睾丸DLBCL）。所有患者均经病理组织活检和免疫组织化学染色确诊，并按照WHO 2016分类标准[8]进行病理复核。

2. 治疗方案：68例睾丸DLBCL患者中，54例（79％）在化疗前接受了睾丸切除术。62例（91％）接受以R-CHOP（利妥昔单抗+环磷酰胺+阿霉素+长春新碱+泼尼松）方案为基础的化疗方案，化疗后常规接受4次鞘内注射（阿糖胞苷50 mg+甲氨蝶呤10 mg+地塞米松5 mg）及对侧睾丸放射治疗（25～30 Gy）。

3. 疗效评价及分期：化疗结束后进行评估，评估手段包括PET-CT（58例，87％）或颈部、胸部、腹部、盆腔增强CT（9例，13％）。按照2014年卢加诺（Lugano）标准[9]进行疗效评估，包括完全缓解（CR）、部分缓解（PR）、疾病稳定（SD）、疾病进展（PD），总有效率（ORR）＝CR率+PR率。按照Ann Arbor分期标准进行临床分期。应用国际预后指数（IPI）对患者进行危险分层[6]。

4. 随访：采用门诊及电话的方式进行随访。随访截止时间为2022年4月15日。中位随访时间为43.8（0.9～167.1）个月。总生存（OS）时间为自诊断之日起至任何原因导致死亡或随访终止的时间。无进展生存（PFS）时间为自诊断之日起至首次发现肿瘤进展、患者死亡或随访终止的时间。

5. 靶向测序检测基因突变：应用组织gDNA提取试剂盒（美国Promega公司产品）提取gDNA。取1 µg DNA制备DNA全基因组文库。使用PCR引物扩增目的基因（55个淋巴瘤相关基因，分别为ARID1A、ATM、B2M、BCL6、BTG1、BTG2、CARD11、CCND3、CD58、CD70、CD79A、CD79B、CIITA、CREBBP、DDX3X、DTX1、DUSP2、EBF1、EP300、EZH2、FAS、FBXW7、FOXO1、GNA13、HIST1H1C、HIST1H1E、IRF4、IRF8、KMT2C、KMT2D、LYN、MAPK7、MPEG1、MTOR、MYC、MYD88、NFKBIE、NOTCH1、NOTCH2、PIM1、PRDM1、PTPN6、SGK1、SOCS1、STAT3、STAT6、TBL1XR1、TET2、TMSB4X、TNFAIP3、TNFRSF14、TP53、TSC2、ZFP36L1、ZNF608），将目标区域DNA富集后，采用Novaseq（美国Illumina公司产品）测序平台进行测序。测序后原始数据利用CCDS、人基因组数据库（HG19）、dbSNP（v138）、1000 genomes、COSMIC、PolyPhen、SIFT等数据库进行生物信息学分析，确定致病基因突变位点。

6. 统计学处理：所有统计学分析均采用SPSS 23.0软件完成。计数资料以例数（百分比）表示，计量资料用中位数（范围）或*x*±*s*表示。组间比较采用*χ*^2^检验及Fisher确切概率法。采用Kaplan-Meier法计算PFS和OS时间。采用Log-rank检验进行单因素分析，Cox比例风险回归模型进行多因素分析。*P*<0.05为差异有统计学意义。

## 结果

1. 临床病理特征：68例睾丸DLBCL患者的中位发病年龄为64（22～84）岁。40例（59％）以阴囊和（或）睾丸肿大为疾病首发症状；23例（34％）患者存在≥2处的多发结外受累；26例（38％）患者乳酸脱氢酶（LDH）水平升高；根据Ann Arbor临床分期，30例（44％）患者为Ⅲ～Ⅳ期；23例（34％）患者IPI评分为中高危/高危组；7例（10％）美国东部肿瘤协作组体力状况（ECOG）评分≥2分；11例（16％）患者合并B症状；54例（79％）行睾丸切除术进行治疗。生发中心来源（GCB）患者11例（21％），非GCB（non-GCB）患者42例（79％），BCL2及MYC蛋白双表达（DE）患者12例（18％）。

68例睾丸DLBCL患者中，原发睾丸DLBCL 45例（66％），继发睾丸DLBCL 23例（34％）。两组年龄>60岁患者比例、细胞起源、DE患者比例的差异均无统计学意义（*P*值均>0.05）。继发组Ann Arbor分期Ⅲ～Ⅳ期（*P*<0.001）、IPI评分3～5分（*P*<0.001）、LDH升高（*P*<0.001）、ECOG评分≥ 2分（*P*＝0.005）患者的比例高于原发组，进行手术治疗的患者比例低于原发组（*P*＝0.038）（表1）。

**表1:** 原发和继发睾丸弥漫大B细胞淋巴瘤患者的临床特征比较［例（％）］

临床特征	原发组（45例）	继发组（23例）	*χ*^2^值	*P*值
年龄			0.400	0.527
≤60岁	13（28.9）	5（21.7）		
>60岁	32（71.1）	18（78.3）		
Ann Arbor分期			44.024	<0.001
Ⅰ～Ⅱ	38（84.4）	0（0）		
Ⅲ～Ⅳ	7（15.6）	23（100）		
LDH升高			14.446	<0.001
否	35（77.8）	7（30.4）		
是	10（22.2）	16（69.6）		
ECOG评分			–	0.005（Fisher检验）
0～1分	44（97.8）	17（73.9）		
≥2分	1（2.2）	6（26.1）		
IPI评分			51.304	<0.001
0～2分	43（95.6）	2（8.7）		
3～5分	2（4.4）	21（91.3）		
细胞起源^a^			0.002	0.968
GCB	7（20.6）	4（21.1）		
non-GCB	27（79.4）	15（78.9）		
DE			0.400	0.527
否	38（84.4）	18（78.3）		
是	7（15.6）	5（21.7）		
手术		4.283	0.038
否	6（13.3）	8（34.8）		
是	39（86.7）	15（65.2）		

**注** LDH：乳酸脱氢酶；ECOG评分：美国东部肿瘤协作组体力状况评分；IPI：国际预后指数；GCB：生发中心来源；non-GCB：非生发中心来源；DE：BCL2及MYC蛋白双表达；^a^共53例患者进行了细胞起源分类

2. 疗效评估与结局评价：68例患者中，1例未进行疗效评估，67例（98.5％）可评估疗效。其中，53例（78％）获得CR，3例（4％）获得PR，ORR为83.6％。45例原发患者中，44例可评估疗效，40例（91％）获得CR，1例（2％）获得PR，ORR为93％。23例继发患者均可评估疗效，13例（57％）获得CR，2例（9％）获得PR，ORR为65％。原发睾丸DLBCL患者的CR率和ORR高于继发睾丸DLBCL患者，差异均有统计学意义（*P*值分别为0.003和0.010）。67例可评估疗效的睾丸DLBCL患者中，29例（43％）出现进展或复发，其中2例（3％）分别在初诊后4个月和22个月出现中枢神经系统进展或复发。

3. 生存分析：睾丸DLBCL患者预期5年PFS率和OS率分别为50.1％和70.8％。原发患者的预期5年PFS率（68.1％对16.5％，*P*<0.001）和预期5年OS率（74.9％对63.4％，*P*＝0.008）均高于继发睾丸DLBCL患者（图1）。

**图1:**
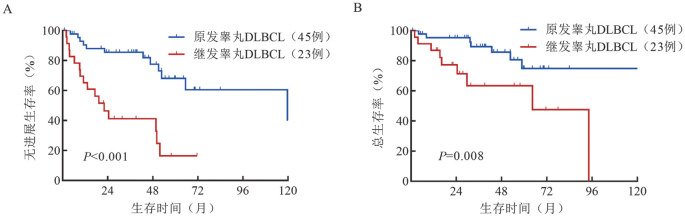
原发和继发睾丸弥漫大B细胞淋巴瘤（DLBCL）患者的无进展生存（A）和总生存（B）曲线

4. 预后因素分析：对68例睾丸DLBCL患者进行单因素分析显示，ECOG评分≥2分（PFS：*P*＝0.018；OS：*P*<0.001），Ann Arbor分期Ⅲ～Ⅳ期（PFS：*P*<0.001；OS：*P*＝0.018）（图2）、LDH升高（PFS：*P*＝0.015；OS：*P*＝0.006）、结外累及数目≥2（PFS：*P*<0.001；OS：*P*＝0.013）是睾丸DLBCL的不良预后因素（表2）。多因素Cox分析未显示独立预后因素。

**图2:**
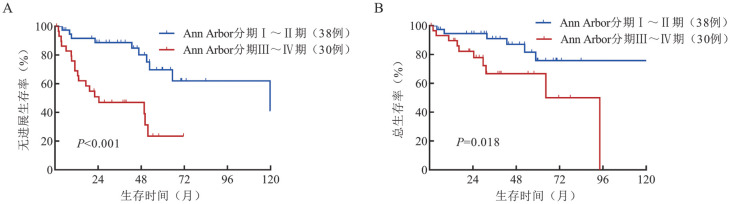
睾丸弥漫大B细胞淋巴瘤患者Ann Arbor分期对无进展生存（A）和总生存（B）的影响

**表2:** 影响睾丸弥漫大B细胞淋巴瘤（DLBCL）患者预后的单因素分析

因素	无进展生存		总生存	
	*HR*（95%*CI*）	*P*值	*HR*（95%*CI*）	*P*值
年龄>60（岁）	0.915（0.403～2.079）	0.831	1.141（0.367～3.547）	0.820
ECOG评分≥2分	3.053（1.148～8.116）	0.018	6.516（2.210～19.209）	<0.001
Ann Arbor分期Ⅲ～Ⅳ期	4.322（1.898～9.843）	<0.001	3.262（1.165～9.136）	0.018
血清LDH升高	2.459（1.163～5.200）	0.015	3.784（1.362～10.518）	0.006
结外累及数目≥2	4.047（1.867～8.774）	<0.001	3.299（1.216～8.951）	0.013
non-GCB	3.844（0.890～16.599）	0.052	1.454（0.312～6.772）	0.632
DE	1.750（0.736～4.160）	0.199	0.794（0.178～3.532）	0.761

**注** ECOG评分：美国东部肿瘤协作组体力状况评分；LDH：乳酸脱氢酶；non-GCB：非生发中心来源；DE：BCL2及MYC蛋白双表达

5. 基因突变分析：68例患者中，20例（14例为原发睾丸DLBCL，6例为继发睾丸DLBCL）进行了靶向测序，共检测出35个基因发生了突变。突变频率大于20％的突变基因为PIM1（12例，60％），MYD88（11例，55％），CD79B（9例，45％），CREBBP（5例，25％），KMT2D（5例，25％），ATM（4例，20％），BTG2（4例，20％）（图3）。继发睾丸DLBCL患者KMT2D的突变率高于原发睾丸DLBCL患者（66.7％对7.1％，*P*＝0.014），且KMT2D突变与睾丸DLBCL患者较低的PFS率相关（*P*＝0.019）（图4）。

**图3:**
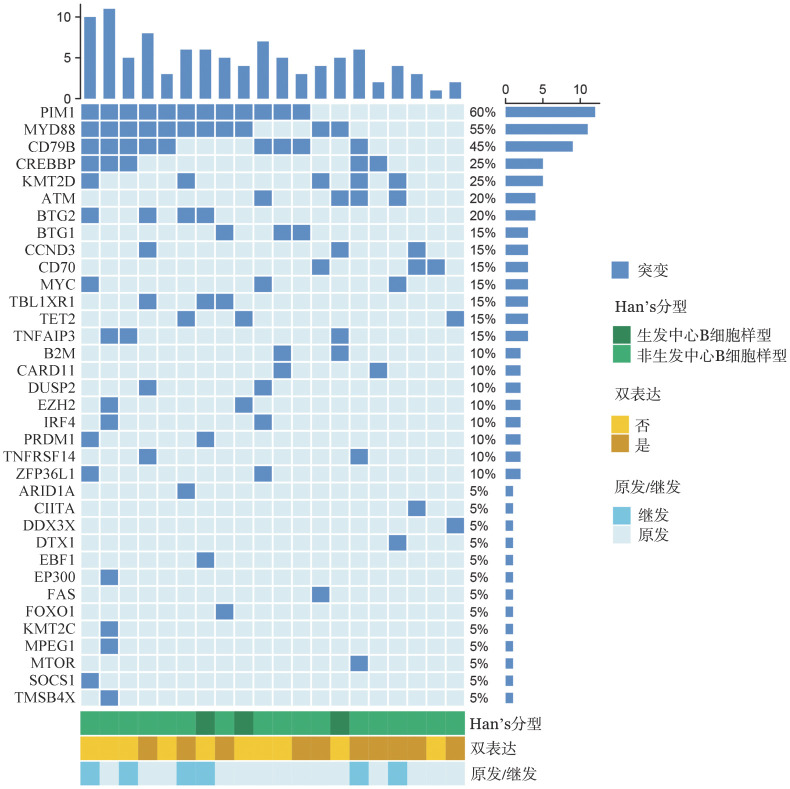
睾丸弥漫大B细胞淋巴瘤患者的基因突变图谱

**图4:**
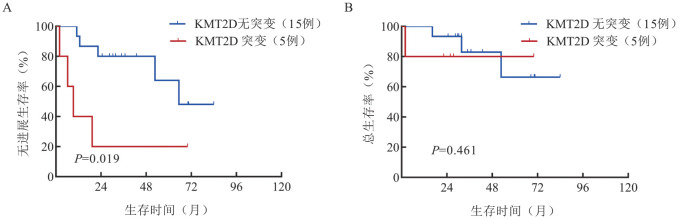
睾丸弥漫大B细胞淋巴瘤患者KMT2D基因突变状态对无进展生存（A）和总生存（B）的影响

## 讨论

睾丸DLBCL是一种罕见的侵袭性结外NHL，好发于老年男性[4]，主要表现为阴囊和（或）睾丸无痛性进行性肿大[2]，多为non-GCB亚型[10]。本研究患者中位发病年龄64（22～84）岁，多以阴囊和（或）睾丸肿大为疾病首发症状（59％），以non-GCB亚型（79％）居多，与文献报道相符[4],[11]–[12]。

睾丸DLBCL可分为原发睾丸DLBCL和继发睾丸DLBCL两类[3]–[4],[11]–[12]。原发睾丸DLBCL多见，且Ann Arbor分期多为Ⅰ～Ⅱ期[3]–[4],[11]–[12]。本研究以原发睾丸DLBCL患者居多（66.2％），且原发患者的Ann Arbor分期Ⅲ～Ⅳ期、IPI评分3～5分、ECOG评分≥2分、LDH升高的比例均低于继发睾丸DLBCL患者。

目前国际公认的睾丸DLBCL一线治疗为基于R-CHOP或R-CHOP样方案化疗的综合治疗方案[7],[13]。一线治疗后，推荐应用甲氨蝶呤鞘内注射及对侧睾丸放疗（25～30 Gy）预防中枢神经系统和对侧睾丸的复发[6]。本研究中，大多数患者接受了手术治疗（54例，79％）及R-CHOP方案化疗（62例，91％），并在化疗后常规接受鞘内注射及对侧睾丸放射治疗。预期5年PFS率和5年OS率分别为50.1％和70.8％。与既往研究显示的睾丸DLBCL患者5年PFS率30％～50％、5年OS率45％～65％相比有所改善[4],[11]–[12]。出现继发中枢神经系统进展或复发的患者比例接近或略低于既往研究[5],[14]，可能的原因包括：①R-CHOP方案已被证实可显著改善睾丸DLBCL患者的PFS率、OS率，减少累积复发率[15]，本研究睾丸DLBCL患者接受R-CHOP或R-CHOP样方案化疗的比例高于既往研究[4],[12]；②有研究表明，睾丸放射治疗可显著降低复发风险[16]–[17]，鞘内注射或可改善睾丸DLBCL患者生存情况[16]，而本研究中睾丸DLBCL患者化疗后常规接受鞘内注射及对侧睾丸放射治疗，比例高于既往研究[4],[11]–[12]；③本研究中位随访时间较既往研究缩短，中枢神经系统进展或复发有待延长随访时间进一步观察。在本研究中，原发睾丸DLBCL患者的CR率、PFS率和OS率均优于继发睾丸DLBCL患者，考虑与继发患者分期较晚、IPI评分较高等有关。本研究发现睾丸DLBCL的不良预后因素包括ECOG评分≥2分、LDH升高、Ann Arbor分期Ⅲ～Ⅳ期和多结外受累，与既往报道一致[12],[18]–[21]。

二代测序技术在肿瘤筛查、诊断、预后等方面具有重要意义。本研究应用靶向测序技术对20例睾丸DLBCL患者肿瘤组织样本中与淋巴瘤相关的55个基因突变状态进行了检测[22]–[23]。B细胞受体介导通路相关基因（PIM1、MYD88、CB79B）在睾丸DLBCL患者中具有较高突变率。Wright等[24]报道的DLBCL分子分型中，MCD亚型以PIM1、MYD88、CD79B基因的高频突变为特征，该亚型易发生结外受累，且睾丸为常见受累部位。伊布替尼联合R-CHOP方案在MCD亚型的DLBCL患者中显示出较好的疗效，无事件生存率和OS率较采用单一R-CHOP方案治疗患者明显升高[25]。故加用BTK抑制剂或可改善睾丸DLBCL患者的预后。此外，KMT2D在本研究中突变率较高（5例，25％），与既往研究相符[26]。继发睾丸DLBCL中KMT2D突变发生率较原发睾丸DLBCL升高（66.7％对7.1％），与睾丸DLBCL患者较低的5年PFS率相关。可部分解释继发睾丸DLBCL预后较差的临床特点，可进一步行大样本队列研究验证。

综上所述，睾丸DLBCL是一种罕见的侵袭性结外NHL，原发睾丸DLBCL的生存情况优于继发睾丸DLBCL。ECOG评分≥2分、Ann Arbor分期Ⅲ～Ⅳ期、LDH升高和多结外受累是睾丸DLBCL患者的不良预后因素。PIM1、MYD88和CD79B基因高频突变或将为睾丸DLBCL的治疗提供新的思路。BTK抑制剂联合化疗或可改善睾丸DLBCL患者的预后。

## References

[1] Nogai H, Dörken B, Lenz G (2011). Pathogenesis of non-Hodgkin's lymphoma[J]. J Clin Oncol.

[2] Pollari M, Leivonen SK, Leppä S (2021). Testicular diffuse large B-cell lymphoma-clinical, molecular, and immunological features[J]. Cancers (Basel).

[3] Cheah CY, Wirth A, Seymour JF (2014). Primary testicular lymphoma[J]. Blood.

[4] Deng L, Xu-Monette ZY, Loghavi S (2016). Primary testicular diffuse large B-cell lymphoma displays distinct clinical and biological features for treatment failure in rituximab era: a report from the International PTL Consortium[J]. Leukemia.

[5] 聂 宝, 黄 欣, 刘 校龙 (2015). 65例睾丸非霍奇金淋巴瘤的临床病理学特征[J]. 中华血液学杂志.

[6] Zhu J, Ma J (2021). Chinese Society of Clinical Oncology (CSCO) diagnosis and treatment guidelines for malignant lymphoma 2021 (English version)[J]. Chin J Cancer Res.

[7] Vitolo U, Seymour JF, Martelli M (2016). Extranodal diffuse large B-cell lymphoma (DLBCL) and primary mediastinal B-cell lymphoma: ESMO Clinical Practice Guidelines for diagnosis, treatment and follow-up[J]. Ann Oncol.

[8] Swerdlow SH, Campo E, Pileri SA (2016). The 2016 revision of the World Health Organization classification of lymphoid neoplasms[J]. Blood.

[9] Cheson BD, Fisher RI, Barrington SF (2014). Recommendations for initial evaluation, staging, and response assessment of Hodgkin and non-Hodgkin lymphoma: the Lugano classification[J]. J Clin Oncol.

[10] Al-Abbadi MA, Hattab EM, Tarawneh MS (2006). Primary testicular diffuse large B-cell lymphoma belongs to the nongerminal center B-cell-like subgroup: A study of 18 cases[J]. Mod Pathol.

[11] Gundrum JD, Mathiason MA, Moore DB (2009). Primary testicular diffuse large B-cell lymphoma: a population-based study on the incidence, natural history, and survival comparison with primary nodal counterpart before and after the introduction of rituximab[J]. J Clin Oncol.

[12] Zucca E, Conconi A, Mughal TI (2003). Patterns of outcome and prognostic factors in primary large-cell lymphoma of the testis in a survey by the International Extranodal Lymphoma Study Group[J]. J Clin Oncol.

[13] National Comprehensive Cancer Network NCCN Clinical Practice Guidelines in Oncology for B-Cell Lymphomas (Version 2. 2023)[DB/OL].

[14] Vitolo U, Chiappella A, Ferreri AJ (2011). First-line treatment for primary testicular diffuse large B-cell lymphoma with rituximab-CHOP, CNS prophylaxis, and contralateral testis irradiation: final results of an international phase II trial[J]. J Clin Oncol.

[15] Kridel R, Telio D, Villa D (2017). Diffuse large B-cell lymphoma with testicular involvement: outcome and risk of CNS relapse in the rituximab era[J]. Br J Haematol.

[16] Mannisto S, Vähämurto P, Pollari M (2019). Intravenous but not intrathecal central nervous system-directed chemotherapy improves survival in patients with testicular diffuse large B-cell lymphoma[J]. Eur J Cancer.

[17] Tokiya R, Yoden E, Konishi K (2017). Efficacy of prophylactic irradiation to the contralateral testis for patients with advanced-stage primary testicular lymphoma: an analysis of outcomes at a single institution[J]. Int J Hematol.

[18] Mazloom A, Fowler N, Medeiros LJ (2010). Outcome of patients with diffuse large B-cell lymphoma of the testis by era of treatment: the M. D. Anderson Cancer Center experience[J]. Leuk Lymphoma.

[19] Lagrange JL, Ramaioli A, Theodore CH (2001). Non-Hodgkin's lymphoma of the testis: a retrospective study of 84 patients treated in the French anticancer centres[J]. Ann Oncol.

[20] Chen B, Cao DH, Lai L (2020). Adult primary testicular lymphoma: clinical features and survival in a series of patients treated at a high-volume institution in China[J]. BMC Cancer.

[21] Cao B, Ji DM, Zhou XY (2011). A clinical analysis of primary testicular diffuse large B-cell lymphoma in China[J]. Hematology.

[22] Zhu Y, Fu D, Shi Q (2022). Oncogenic mutations and tumor microenvironment alterations of older patients with diffuse large B-cell lymphoma[J]. Front Immunol.

[23] Shen R, Xu PP, Wang N (2020). Influence of oncogenic mutations and tumor microenvironment alterations on extranodal invasion in diffuse large B-cell lymphoma[J]. Clin Transl Med.

[24] Wright GW, Huang DW, Phelan JD (2020). A probabilistic classification tool for genetic subtypes of diffuse large B cell lymphoma with therapeutic implications[J]. Cancer Cell.

[25] Wilson WH, Wright GW, Huang DW (2021). Effect of ibrutinib with R-CHOP chemotherapy in genetic subtypes of DLBCL[J]. Cancer Cell.

[26] Guo D, Hong L, Ji H (2022). The mutation of BTG2 gene predicts a poor outcome in primary testicular diffuse large B-cell lymphoma[J]. J Inflamm Res.

